# Curcumin ameliorates neuropathic pain by down-regulating spinal IL-1β via suppressing astroglial NALP1 inflammasome and JAK2-STAT3 signalling

**DOI:** 10.1038/srep28956

**Published:** 2016-07-06

**Authors:** Shenbin Liu, Qian Li, Meng-Ting Zhang, Qi-Liang Mao-Ying, Lang-Yue Hu, Gen-Cheng Wu, Wen-Li Mi, Yan-Qing Wang

**Affiliations:** 1 Department of Integrative Medicine and Neurobiology, The Academy of Integrative Medicine, School of Basic Medical Sciences, Institutes of Brain Science, Brain Science Collaborative Innovation Center, State Key Laboratory of Medical Neurobiology, Fudan University, Shanghai, 200032, China

## Abstract

Curcumin has been shown to possess strong anti-inflammatory activity in many diseases. It has been demonstrated that the janus kinase 2 (JAK2)-signal transducer and activator of transcription 3 (STAT3) cascade and the NAcht leucine-rich-repeat protein 1 (NALP1) inflammasome are important for the synthesis of Pro-Interleukin (IL)-1β and the processing of the inactive protein to its mature form, which plays an active role in the pathogenesis of neuropathic pain. The present study showed that repeated intraperitoneal injection of curcumin ameliorated SNI-induced mechanical and cold allodynia in a dose-dependent manner and inhibited the elevation of spinal mature IL-1β protein levels. Additionally, repeated curcumin treatment significantly inhibited the aggregation of the NALP1 inflammasome and the activation of the JAK2-STAT3 cascade in spinal astrocytes. Furthermore, the genetic down-regulation of NALP1 inflammasome activation by NALP1 siRNA and the pharmacological inhibition of the JAK2-STAT3 cascade by AG490 markedly inhibited IL-1β maturation and Pro-IL-1β synthesis, respectively, and reduced SNI-induced pain hypersensitivity. Our results suggest that curcumin attenuated neuropathic pain and down-regulated the production of spinal mature IL-1β by inhibiting the aggregation of NALP1 inflammasome and the activation of the JAK2-STAT3 cascade in astrocytes.

Curcumin, a major component extracted from the rhizome *Curcuma longa*, has been consumed by humans as a curry spice for centuries. Because it has been shown to possess strong anti-inflammatory activity and to inhibit the production of inflammatory cytokines, curcumin is used for the treatment of various neuroinflammatory and neurodegenerative conditions of the central nervous system (CNS) such as Alzheimer’s disease, major depression, and diabetic neuropathy^1^. Recently, several studies demonstrated an anti-nociceptive effect of curcumin in neuropathic pain and inflammatory pain^2^,^3^. However, its exact mechanism of action is not fully understood.

As a vital pro-inflammatory cytokine, interleukin (IL)-1β has been shown to actively participate in the pathogenesis of neuropathic pain and to induce a secondary injury cascade during the course of a disease^4^. Previous studies demonstrated that the exogenous intrathecal administration of IL-1β induced obvious pain behaviours^5^, whereas the blockade of spinal IL-1β signalling relieved nerve injury-induced neuropathic pain^6^, supporting a crucial role for spinal IL-1β in the development of neuropathic pain. Recent studies demonstrated that curcumin is a highly pleiotropic molecule that interacts with numerous molecular targets^7^. However, the effect of curcumin on spinal IL-1β production in neuropathic pain remains unclear.

A two-signal model has been proposed to explain the regulation of IL-1β production^8^,^9^,^10^. One signal is for the expression of the Pro-IL-1β gene, which is mediated by the induction of cellular signalling pathways (e.g., the JAK2-STAT3 pathway) via ligands for Toll-like receptors, whereas the second signal involves the inflammasome oligomerization (e.g., NAcht leucine-rich repeat protein 1 (NALP1) inflammsome), which results in the caspase-1-dependent cleavage of Pro-IL-1β and the release of the biologically active, mature IL-1β. Our previous study reported that the spinal NALP1 inflammasome, which is composed of the NALP1 protein, caspase-1, and the adaptor protein apoptosis-associated speck-like protein containing a caspase-activating recruitment domain (ASC), might participate in IL-1β maturation and contribute to the development of neuropathic pain^11^. These results led us to question whether IL-1β and the NALP1 inflammasome are responsible for the analgesic effect of curcumin.

The present study was designed to determine the effect of repeated curcumin treatment on spared nerve injury (SNI)-induced neuropathic pain and to investigate the mechanism by which it stimulates the production of the bioactive, mature IL-1β in the mouse spinal cord. Our present study demonstrated for the first time that curcumin potently inhibits the JAK2-STAT3 cascade and the NALP1 inflammasome. In doing so it inhibits Pro-IL-1β expression and IL-1β maturation, resulting in the management of neuropathic pain.

## Results

### Effect of the repeated intraperitoneal injection of curcumin on mechanical and cold allodynia in SNI mice

**Figure 1:**
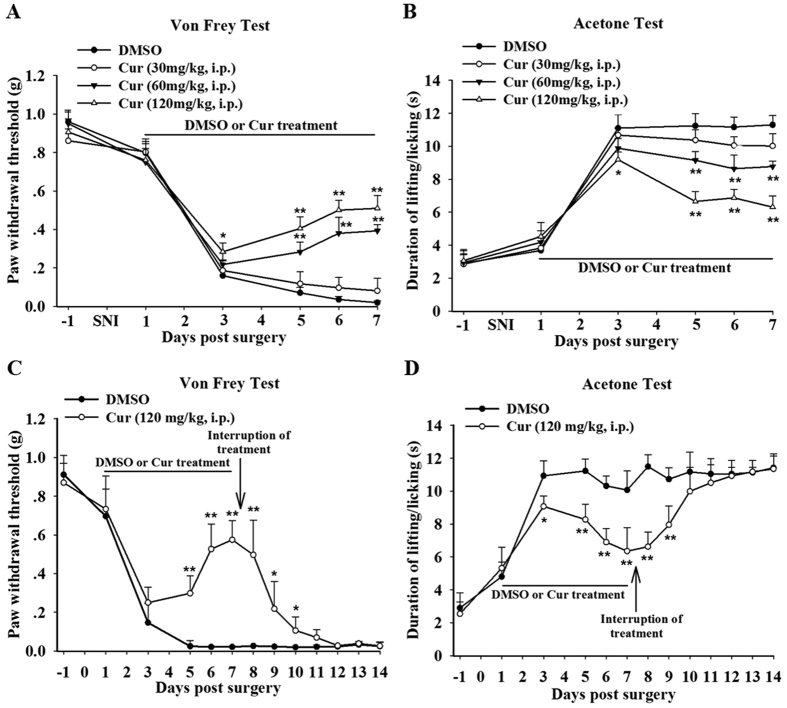
Anti-allodynic effect of curcumin on SNI-induced neuropathic pain. Time course showing the changes in the mechanical withdrawal threshold in the Von Frey test (**A**) and the duration of lifting/licking in the acetone test (**B**) in the ipsilateral hind paw of mice chronically intraperitoneal (i.p) treated with vehicle (20% DMSO) or curcumin (30 mg/kg, 60 mg/kg, or 120 mg/kg). Treatment started on day 1 after SNI and was administered twice a day until day 7. *p < 0.05 and **p < 0.01 compared with the DMSO treatment group; two-way ANOVA followed by Bonferroni *post hoc* test; n = 8 per group. (**C,D**) Effect of curcumin treatment (120 mg/kg, i.p) on the response to mechanical or cold stimuli in SNI mice. The administration of vehicle or curcumin was stopped after 7 days of repeated treatment. *p < 0.05 and **p < 0.01 compared with the DMSO group; one-way ANOVA followed by Bonferroni *post hoc* test; n = 8 per group. Cur = curcumin. All of the data are shown as the mean ± standard error of the mean (SEM).

Neuropathic pain in mice can be examined by measuring the paw withdrawal threshold in the Von Frey test (mechanical allodynia) and the duration of paw lifting/licking in the acetone test (cold allodynia)^12^,^13^. A significant reduction in the mechanical threshold and increase in the duration of lifting/licking was observed in mice treated with vehicle (20% DMSO) for 7 days after SNI, indicating a sustained SNI-induced mechanical and cold allodynia (Fig. 1A,B). To examine the analgesic effect of curcumin, different doses of curcumin or vehicle were administered intraperitoneally (i.p) twice daily from day 1 until day 7 after surgery (SNI and Sham). As shown in Fig. 1A,B, repeated treatment with curcumin effectively and dose-dependently alleviated SNI-induced pain hypersensitivity. For mechanical allodynia (Fig. 1A), an analysis by two-way ANOVA revealed a significant effect of Treatment (F_3,196_ = 36.79 and P < 0.001), Time (F_7,196_ = 59.80 and P < 0.001), and Time × Treatment (F_21,196_ = 9.57 and P < 0.001). The Bonferroni post hoc tests showed that curcumin at a dose of 120 mg/kg did not affect paw withdrawal threshold in the first day. However, an attenuation of mechanical allodynia appeared at day 3 and persisted until day 7 after SNI. Curcumin at 60 mg/kg reduced SNI-induced mechanical allodynia from day 5 until day 7 after SNI (Fig. 1A). Curcumin also attenuated SNI-induced cold allodynia (Fig. 1B, Treatment, F_3,196_ = 65.98 and P < 0.001; Time, F_7,196_ = 68.34 and P < 0.001; and Time × Treatment, F_21,196_ = 10.30 and P < 0.01). The Bonferroni post hoc tests showed that curcumin at a dose of 120 mg/kg reversed SNI-induced cold allodynia from day 3 to day 7 after SNI. Curcumin at the dose of 60 mg/kg also diminished SNI-induced cold allodynia from day 5 until day 7 (Fig. 1B). The lowest dose of curcumin (30 mg/kg) did not significantly influence mechanical and cold allodynia at any time point (Fig. 1A,B).

We also evaluated the effect of the withdrawal of repeated curcumin treatment on the pain threshold. As shown in Fig. 1C,D, when curcumin administration was stopped following 7 days of treatment, the anti-nociceptive effect was maintained for at least two more days but was followed by a relapse of mechanical and cold allodynia in SNI mice.

Collectively, these data demonstrated that repeated curcumin treatment attenuated mechanical and cold allodynia following SNI-induced neuropathic pain.

### Effect of repeated curcumin treatment on spinal Pro-IL-1β production and IL-1β maturation in SNI mice

**Figure 2:**
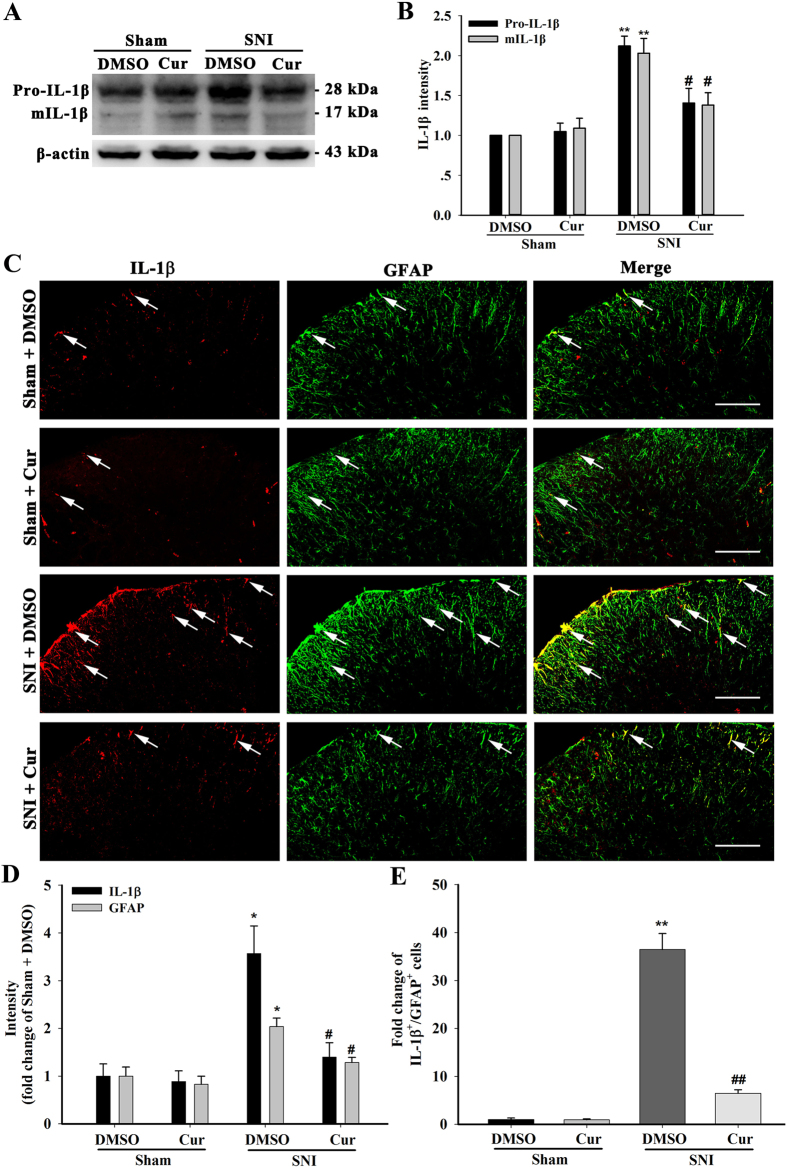
Effect of repeated curcumin (120 mg/kg, i.p) treatment on the expression of spinal IL-1β in SNI mice. (**A**) Western blot showing the expression of Pro-IL-1β and mature IL-1β (mIL-1β) in the spinal cord of repeated DMSO- or curcumin-treated mice on day 7 after SNI or sham surgery. Cropped gels/blots are used in this figure and the immunoblots were obtained from the microgel running in the same experimental conditions. (**B**) Quantification of the Pro-IL-1β and mIL-1β bands. **p < 0.01 compared with the sham + DMSO group; ^#^p < 0.01 compared with the SNI + DMSO group; one-way ANOVA followed by Bonferroni *post hoc* test; n = 4 per group. (**C**) IL-1β and GFAP double staining in the spinal dorsal horn of repeated DMSO- and curcumin-treated mice day 7 after sham and SNI surgery. The arrows indicate the double-labelled cells. (**D**) Intensity of IL-1β and GFAP staining in the superficial dorsal horn (laminae I–III). *p < 0.05, compared with the Sham + DMSO group; _#_p < 0.05 compared with the SNI + DMSO group; one-way ANOVA followed by Bonferroni *post hoc* test; n = 3–4 per group. (**F**) Number of IL-1β^+^/GFAP^+^ double-labelled cells in the superficial dorsal horn (laminae I–III). *p < 0.05, compared with the Sham + DMSO group; ^#^p < 0.05 compared with the SNI + DMSO group; one-way ANOVA followed by Bonferroni *post hoc* test; n = 3–4 per group. Scale bars = 50 μm.

Emerging evidence indicates that spinal neuroinflammation and immune responses that result from nerve damage and lead to the up-regulation of pro-inflammatory cytokines contribute to the development of neuropathic pain^14^. Many studies have reported that the pivotal spinal pro-inflammatory cytokine IL-1β contributes to the development and maintenance of neuropathic pain^15^. To assess the effect of curcumin on spinal IL-1β production in response to neuropathic pain, we investigated IL-1β expression on the seventh day after repeated curcumin (i.p, 120 mg/kg, twice daily from day 1 until day 7 after surgery) treatment in SNI mice. Western blotting showed that the upregulation of spinal Pro-IL-1β (One-way ANOVA, F_3,12_ = 4.86, p < 0.05) and mature IL-1β (mIL-1β) (One-way ANOVA, F_3,12_ = 5.56, p < 0.05) was significantly diminished in curcumin-treated mice compared with DMSO-treated mice 7 days after SNI (Fig. 2A,B). Immunofluorescence results also revealed that compared with the Sham + DMSO group, the intensity of IL-1β-immunoreactivity (IR) and GFAP-IR and the number of IL-1β^+^/GFAP^+^ cells were significantly increased in the spinal cord of SNI mice, while curcumin treatment markedly inhibited these upregulation (Fig. 2C–E). These results suggest that repeated curcumin treatment reduced the availability of spinal Pro-IL-1β availability and the maturation of IL-1β.

### Role of spinal NALP1 in SNI-induced neuropathic pain and IL-1β maturation

**Figure 3:**
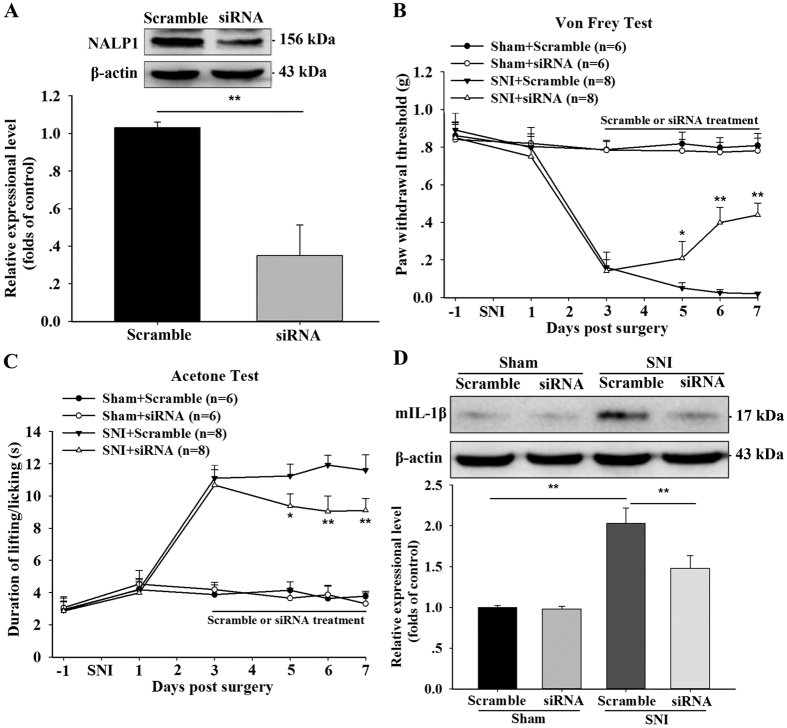
Role of spinal NALP1 in SNI-induced neuropathic pain. (**A**) Immunoblotting showed that the small interfering RNA against NALP1 (siRNA) (5 μg), but not the scrambled control (Scramble), reduced NALP1 protein levels in the spinal cords dissected from SNI mice on day 7 after behaviour analysis. Cropped gels/blots are used in this figure and the immunoblots were obtained from the microgel running in the same experimental conditions. **p < 0.01; student’s t test; n = 4 per group. (**B,C**) Time course of the effect of NALP1 siRNA on SNI-induced mechanical and cold allodynia. The repeated intrathecal injection of siRNA targeting NALP1 (5 μg), but not scrambled siRNA, from day 3 to day 7 after SNI, facilitated recovery from SNI-induced mechanical and cold allodynia. siRNA administration did not affect the baseline of mechanical threshold and lifting latency in sham mice. *p < 0.05 and **p < 0.01 compared with the SNI + scrambled group; two-way ANOVA followed by Bonferroni *post hoc* test; n = 8 per group. (**D**) 5 μg NALP1 siRNA, but not scrambled siRNA, significantly reversed the SNI-induced up-regulation of mature IL-1β as determined by western blotting. Cropped gels/blots are used in this figure and the immunoblots were obtained from the microgel running in the same experimental conditions. **p < 0.01; one-way ANOVA followed by Bonferroni *post hoc* test; n = 4 per group. All of the data are shown as the mean ± SEM.

To further examine the potential role of the NALP1 inflammasome in pain modulation, we investigated the role of spinal NALP1, which is the scaffold protein of NALP1 inflammasome, in SNI-induced neuropathic pain. The intrathecal injection of small interfering RNA (siRNA) targeting NALP1 in the spinal cord of SNI mice administered from day 3 until day 7 significantly reduced NALP1 protein levels compared with the scrambled control siRNA (Student *t*-test, t = 4.11, p < 0.01; Fig. 3A). The paw withdrawal threshold in the Von Frey test was markedly increased, and the duration of lifting/licking of the paw in the acetone test was markedly decreased in mice treated with NALP1 siRNA compared with the responses in the mice treated with the scrambled siRNA (Fig. 3B,C). However, the siRNA targeting NALP1 had no effect on the basal response of the sham mice in the Von Frey and acetone tests (p > 0.05, Fig. 3B,C). Western blotting revealed that the SNI mice treated with NALP1 siRNA also displayed a significant reduction in the level of mature IL-1β compared with the scrambled siRNA group (One-way ANOVA, F_3,12_ = 4.69, p < 0.05, Fig. 3D,E). Thus, endogenous spinal NALP1 may contribute to the response to neuropathic pain by accelerating IL-1β maturation and nociceptive transmission.

### Effect of curcumin on the SNI-induced aggregation of the spinal NALP1 inflammasome

**Figure 4:**
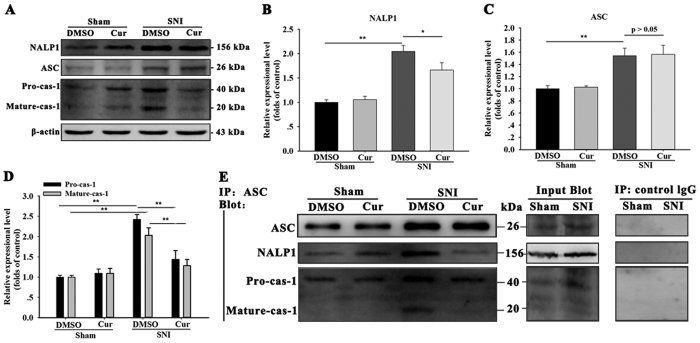
Effect of repeated curcumin treatment on the activation of the spinal NALP1 inflammasome in SNI mice. (**A–D**) SNI injury resulted in the increased expression of NALP1, ASC, and Pro- and mature-caspase-1 in the spinal cord on day 7 after SNI. The SNI-induced elevation in NALP1 and Pro- and mature-caspase-1 expression was significantly reduced after repeated curcumin treatment. *p < 0.05 and **p < 0.01; one-way ANOVA followed by Bonferroni *post hoc* test; n = 4 per group. All of the data are shown as the mean ± SEM. (**E**) ASC immunoprecipitation from lysates obtained from SNI- or sham-treated mice that received repeated treatment with curcumin (120 mg/kg) or DMSO. Curcumin reversed the SNI-induced aggregation of the NALP1 inflammasome proteins. Cropped gels/blots are used in this figure and the immunoblots were obtained from the microgel running in the same experimental conditions.

**Figure 5:**
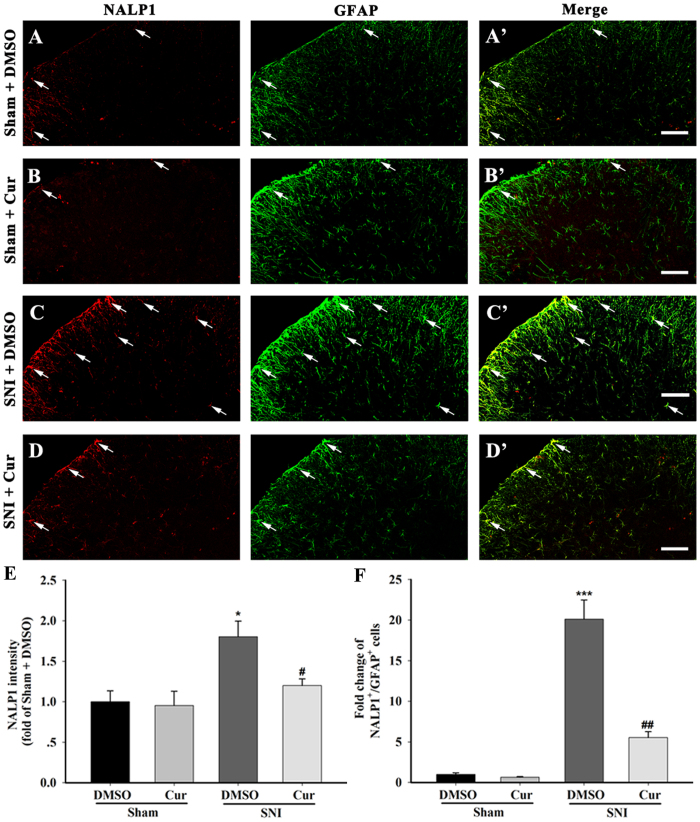
NALP1 and GFAP double staining in the spinal dorsal horn of mice chronically treated with DMSO or curcumin (120 mg/kg) on the day 7 after sham or SNI surgery. The sham surgery induced a weak NALP1-IR in the ipsilateral spinal dorsal horn (**A,B**). Changes in the NALP1-IR were not observed in the sham group after intraperitoneal curcumin administration (**A,B**). After SNI surgery, increased NALP1-IR and GFAP-IR were detected ipsilateral to the lesion of the spinal dorsal horn, and a large part of the NALP1-IRs were co-labelled with GFAP-IR, indicating that NALP1 accumulated in the spinal cord astrocytes on the day 7 after SNI (**C,C**’). The intraperitoneal administration of curcumin inhibited the expression of NALP1-IR in the ipsilateral spinal dorsal horn after SNI (**D**). (**E**) Intensity of NALP1 staining in the superficial dorsal horn (laminae I–III). *p < 0.05, compared with the Sham + DMSO group; ^#^p < 0.05 compared with the SNI + DMSO group; one-way ANOVA followed by Bonferroni *post hoc* test; n = 3–4 per group. (**F**) Number of NALP1^+^/GFAP^+^ double-labelled cells in the superficial dorsal horn (laminae I–III). *p < 0.05, compared with the Sham + DMSO group; ^#^p < 0.05 compared with the SNI + DMSO group; one-way ANOVA followed by Bonferroni *post hoc* test; n = 3–4 per group. Scale bar = 50 μm.

**Figure 6:**
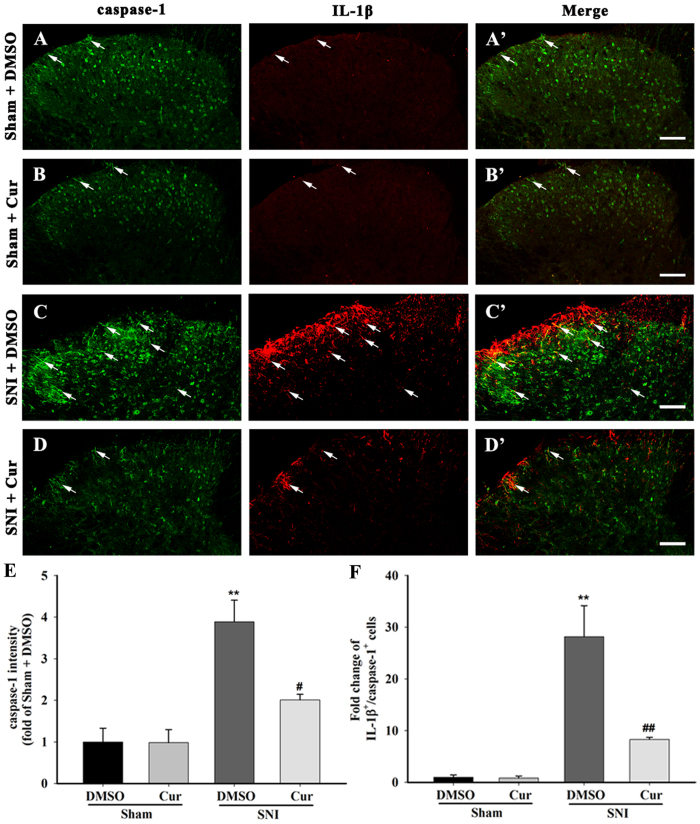
Caspase-1 and IL-1β double staining in the spinal dorsal horn of mice chronically treated with DMSO or curcumin (120 mg/kg) on day 7 after sham or SNI surgery. The sham surgery induced a weak caspase-1-IR in the ipsilateral spinal dorsal horn (**A,B**). The changes in the caspase-1-IR were not observed in the sham group after intraperitoneal repeated curcumin (120 mg/kg) administration (**A,B**). After SNI surgery, increased caspase-1-IR was detected ipsilaterally to the lesion of the spinal dorsal horn, and many of the IL-1β-IR was co-labelled with caspase-1-IRs (**C,C’**). The intraperitoneal administration of curcumin inhibited the expression of caspase-1-IR in the ipsilateral spinal dorsal horn after SNI (**D**). (**E**) Intensity of caspase-1 staining in the superficial dorsal horn (laminae I–III). **p < 0.01, compared with the Sham + DMSO group; ^#^p < 0.05 compared with the SNI + DMSO group; one-way ANOVA followed by Bonferroni *post hoc* test; n = 3–4 per group. (**F**) Number of IL-1β^+^/caspase-1^+^ double-labelled cells in the superficial dorsal horn (laminae I–III). **p < 0.01, compared with the Sham + DMSO group; ^##^p < 0.01 compared with the SNI + DMSO group; one-way ANOVA followed by Bonferroni *post hoc* test; n = 3–4 per group. Scale bar = 50 μm.

To assess whether NALP1 inflammasome caspase-1 platform is involved in the suppression of IL-1β maturation by curcumin, we observed the changes of the component of the NALP1 inflammasome: the scaffold proteins NALP1, the adaptor protein ASC and the effector protein caspase-1 after the curcumin treatment. The SNI mice that received repeated doses of 120 mg/kg curcumin displayed a reduction in up-regulated NALP1 (One-way ANOVA, F_3,12_ = 5.10, p < 0.05), Pro-caspase-1 (One-way ANOVA, F_3,12 _= 6.98, p < 0.05), and mature caspase-1 (One-way ANOVA, F_3,12_ = 4.85, p < 0.05). In contrast, the ASC protein levels in these mice remained unchanged (One-way ANOVA, F_3,12_ = 3.02, p > 0.05) (Fig. 4A–D). In the IP experiments, repeated treatment with 120 mg/kg curcumin reversed the increased aggregation of ASC, NALP1, and caspase-1 within the NALP1 inflammasome complex (Fig. 4E). Additionally, enhanced NALP1- IR (Fig. 5) and caspase-1-IR (Fig. 6) were observed in the ipsilateral spinal dorsal horn on day 7 after SNI, whereas only weak NALP1 and caspase-1 protein levels were observed in the sham mice. Furthermore, almost all of the NALP1-positive cells were co-expressed with GFAP (Fig. 5), and a large proportion of the caspase-1-positive cells would also exhibit IL-1β expression (Fig. 6). Curcumin-treated mice had less NALP1-IR (Fig. 5) and caspase-1-IR (Fig. 6) in the spinal cord than the DMSO-treated group. All these data demonstrates that curcumin inhibited the activation of the SNI-induced NALP1 inflammasome.

### Effect of curcumin on Pro-IL-1β synthesis mediated by the spinal JAK2-STAT3 cascade in SNI mice

**Figure 7:**
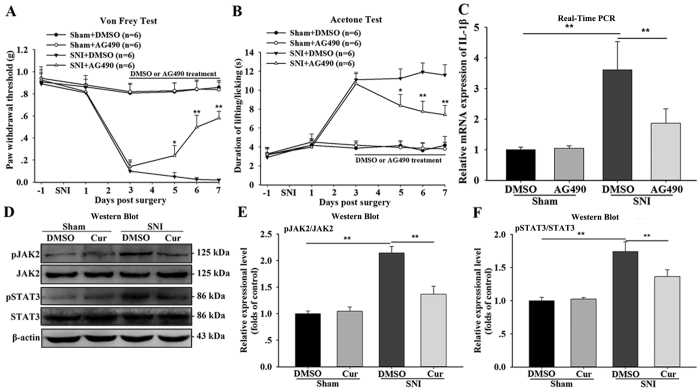
Effect of curcumin on the spinal JAK2-STAT3 cascade in SNI mice. (**A,B**) Mice with peripheral nerve injury were intrathecally injected with AG490 (5 μg) or vehicle (1% DMSO, 5 μl) once a day on days 3–7. The paw withdrawal threshold in response to mechanical stimulation by Von Frey filaments and the duration of lifting/licking of the paw following cold stimulation by acetone were measured in the begining (day 0) and on days 1, 3, 4, 5, 6, and 7 following peripheral nerve injury. *p < 0.05 and **p < 0.01 compared with the SNI +  DMSO group; two-way ANOVA followed by Bonferroni *post hoc* test; n = 6 per group. (**C**) Effect of AG490 on the expression of spinal IL-1β mRNA as assessed by real-time PCR on day 7 after surgery. *p < 0.05 and **p < 0.01; one-way ANOVA followed by Bonferroni *post hoc* test; n = 4 per group. (**D,E**) SNI resulted in the increased expression of pJAK2 and pSTAT3 in the spinal cord on the seventh day after SNI. The SNI-induced elevation of pJAK2 and pSTAT3 was significantly reversed in the spinal cords of mice following the repeated intraperitoneal injection of curcumin (120 mg/kg). Cropped gels/blots are used in this figure and the immunoblots were obtained from the microgel running in the same experimental conditions. *p < 0.05 and **p < 0.01; one-way ANOVA followed by Bonferroni *post hoc* test; n = 4 per group. All of the data are shown as the mean ± SEM.

We next explored the mechanism by which curcumin inhibits the synthesis of spinal Pro-IL-1β. As shown in Fig. 2C, curcumin not only inhibited IL-1β, but also decreased GFAP expression in the spinal dorsal horn. The spinal astroglial JAK2-STAT3 cascade has been shown to attenuate astrocyte proliferation and function during the maintenance of neuropathic pain^16^. In addition, the activated JAK2-STAT3 cascade plays a vital role in IL-1β mRNA expression^17^,^18^. We thus examined whether the spinal JAK2-STAT3 cascade participates in the curcumin-mediated inhibition of IL-1β synthesis. Mice with nerve injury that were treated with AG490 (5 μg, intrathecal injection), a partial inhibitor of the JAK2-STAT3 cascade, from day 3 to day 7 after SNI, showed a significant increase in the paw withdrawal threshold and a reduction in the duration of lifting/licking of the paw (Fig. 7A,B). Real-time PCR revealed that the SNI mice that received AG490 treatment also displayed a remarkable reduction in the expression of spinal IL-1β mRNA compared to the vehicle group (One-way ANOVA, F_3,12_ = 6.102, p < 0.01, Fig. 7C). These results indicate that the spinal astroglial JAK2-STAT3 cascade might contribute to the synthesis of IL-1β mRNA after SNI.

**Figure 8:**
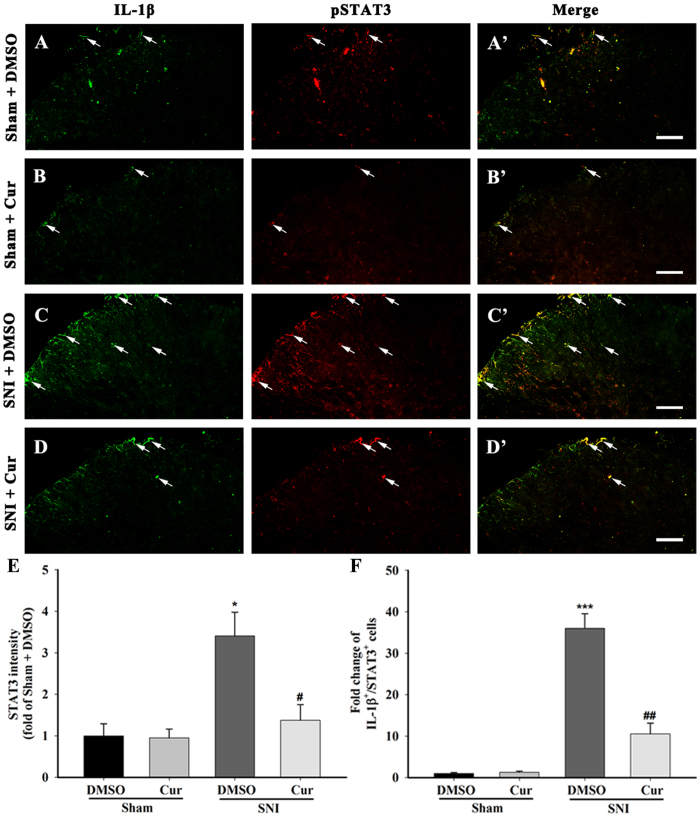
pSTAT3 and IL-1β double staining in the spinal dorsal horn of mice chronically treated with DMSO or curcumin (120 mg/kg) on day 7 after sham or SNI surgery. The sham surgery induced a weak pSTAT3-IR in the ipsilateral spinal dorsal horn (**A,B**). The changes in the pSTAT3-IR were not observed in the sham group after intraperitoneal curcumin administration (**A,B**). After SNI surgery, increased pSTAT3-IR and GFAP-IR were detected ipsilateral to the lesion of the spinal dorsal horn, and many of the IL-1β-IR were co-labelled with pSTAT3-IRs, indicating that pSTAT3 accumulated in spinal cord astrocytes on the day 7 after SNI (**C,C**’). The intraperitoneal administration of curcumin inhibited the expression of pSTAT3-IR in the ipsilateral spinal dorsal horn after SNI (**D**). (**E**) Intensity of STAT3 staining in the superficial dorsal horn (laminae I–III). **p < 0.01, compared with the Sham + DMSO group; ^#^p < 0.05 compared with the SNI + DMSO group; one-way ANOVA followed by Bonferroni *post hoc* test; n = 3–4 per group. (**F**) Number of IL-1β^+^/STAT3^+^ double-labelled cells in the superficial dorsal horn (laminae I–III). ***p < 0.001, compared with the Sham + DMSO group; ^##^p < 0.01 compared with the SNI + DMSO group; one-way ANOVA followed by Bonferroni *post hoc* test; n = 3–4 per group. Scale bar = 50 μm.

On the seventh day after SNI, western blotting showed that the protein expression of spinal activated JAK2 and STAT3 was dramatically increased compared with the sham control group and that this up-regulation was significantly attenuated by repeated curcumin treatment (One-way ANOVA, F_3,12_ = 4.89, p < 0.05, Fig. 7D,E). Additionally, double staining revealed an increased expression and co-localization of pSTAT3-IR and IL-1β-IR on day 7 after SNI (Fig. 8). Again, this up-regulation was significantly reduced after curcumin injection (Fig. 8). Collectively, these data demonstrates that curcumin might reduce the synthesis of Pro-IL-1β after SNI by inhibiting the activation of the spinal astroglial JAK2-STAT3 cascade.

## Discussion

Curcumin has attracted considerable attention for its various bioactivities^19^,^20^. It has been well established that curcumin exerts a variety of peripheral pharmacological activities, especially anti-inflammation^1^. In this study, we focused on the effect of repeated curcumin treatment on SNI-induced neuropathic pain and further investigated its mechanism of action in relation to the production of bioactive, mature IL-1β in the spinal cord. The present study revealed that curcumin attenuates neuropathic pain and down-regulates the production of mature IL-1β in the spinal cord in association with inhibiting the NALP1 inflammasome and the activation of the JAK2-STAT3 pathway in astrocytes.

Previous studies showed that the curcumin and curcumin-loaded PLGA nanoparticles are well absorbed, has good tissue penetration, and readily crosses the blood-brain barrier^21^. Recently, there has been compelling evidence demonstrating the activity of curcumin in the central nervous system. In clinical, curcumin have been used for the treatment of Alzheimer’s disease patients at the dose of 4 g/day for 24 weeks^22^, and also 1 g/day for 30 days for anxiety and depression in obese individuals^23^. In animal models, curcumin has been shown to be involved in neuro-protection, to display antidepressant and anti-nociceptive effects and to reverse impaired cognition and neuronal plasticity^24^,^25^,^26^. Additionally, curcumin have used for the treatment of chronic construction injury- and diabetes-induced neuropathic pain in mice in the dose of 45 mg/kg and 60 mg/kg (twice a day in continuous treatment for 3 to 4 weeks)^3^,^27^. Consistently, in the present study, repeated intraperitoneal treatment with 60 and 120 mg/kg curcumin for 7 consecutive days significantly attenuated SNI-induced mechanical allodynia. However, treatment with 30 mg/kg curcumin showed no significant analgesic effect, which is consistent with the previous findings that^28^ 25 mg/kg curcumin failed to ameliorate formalin-induced orofacial pain.

Studies have indicated that curcumin ameliorates carcinogenesis^29^, colitis^30^, and psoriasis-like inflammation^31^ by down-regulating IL-1β. As a vital pro-inflammatory cytokine, spinal IL-1β has been shown to be involved in the development of neuropathic pain^15^. In the current study, repeated curcumin treatment significantly suppressed SNI-induced spinal IL-1β expression in astrocytes. In agreement with these results, other evidence suggests that the pro-inflammatory cytokine IL-1β is predominantly expressed in astrocytes in the spinal cord^32^,^33^ and that curcumin significantly decreases the expression of IL-1β in cultured spinal primary astrocytes^2^. These studies suggest that the down-regulation of spinal IL-1β by curcumin might be responsible for its anti-allodynic effect. In addition to the inhibition of spinal IL-1β production, several other possible mechanisms have been proposed to explain the anti-nociceptive effect of curcumin. These include the antagonism of TRPV1^34^, the inhibition of TNF-α/NO release^35^, and mechanisms involving the descending monoamine system as well as opioid receptors^3^.

Our study revealed that SNI induced an obvious and significant aggregation of NALP1, caspase-1, and the adaptor protein ASC, which suggests that the NALP1 inflammasome is activated during neuropathic pain. Additionally to our previous study, some other studies have reported that the intrathecal or peripheral injection of the caspase-1 inhibitor Ac-YVAD-CMK significantly attenuated CCI-induced thermal hyperalgesia^11^ and post-traumatic regional nociceptive sensitization^36^, respectively, suggesting that the central and peripheral NALP1 inflammasome might contribute to pain sensitization. Consistently, in the present study, the intrathecal injections of NALP1 siRNA not only significantly down-regulated IL-1β maturation but also attenuated neuropathic pain, which further suggests that the activated spinal NALP1 inflammasome contributes to SNI-induced neuropathic pain. Furthermore, recent studies reported that the peripheral NLRC4 inflammasome components NLRC4 and ASC are involved in the activation of caspase-1 and the maturation of IL-1β and that they contribute to acute inflammatory pain^37^. Whether the central NLRC4 inflammasome participates in the maturation of IL-1β during neuropathic pain still needs to be further investigated.

Previous studies have reported that in murine macrophages, the NALP1 inflammasome can be activated by the anthrax lethal toxin, which so far has been the only toxin identified to activate NALP1^38^. However, the specific SNI-induced stimulus that activates the NALP1 inflammasome in spinal astrocytes remains unclear. Studies have shown that decreased levels of intracellular K^+^ result in the activation of caspase-1 and the release of IL-1β in cultured primary astrocytes and neurons^39^. However, whether K^+^ mediates SNI-induced NALP1 activation needs to be examined further. Curcumin has been shown to protect against ischaemic brain injury by suppressing the activation of the endoplasmic reticulum stress-associated TXNIP/NLRP3 inflammasome^40^. However, the mechanism by which curcumin inhibits the aggregation of the NALP1 inflammasome still needs to be studied. Additionally, our present data showed that curcumin treatment inhibited the expression of NALP1 and caspase-1 but not ASC in the spinal cord of SNI mice. These different regulations by curcumin may be due to the different regulatory mechanisms for the NALP1, caspase-1 and ASC expression. Generally, these results indicate that curcumin might suppress the aggregation of the spinal NALP1 inflammasome, thereby inhibiting IL-1β maturation and neuropathic pain.

Previous studies have reported that the activation of JAK2-STAT3 signalling mediates IL-1β mRNA expression *in vitro* in pancreatic acinar AR42J cells stimulated with cerulein as well as *in vivo* in rats with cerulean-induced pancreatitis^17^. Additionally, the intrathecal injection of AG490 (a JAK2-STAT3 cascade inhibitor) markedly decreased the expression of spinal IL-1β mRNA induced by peripheral inflammation^41^. Consistently, we found that the intrathecal injection of a JAK2-STAT3 cascade inhibitor not only ameliorated neuropathic pain, but also reduced the up-regulation of spinal IL-1β mRNA.

It has been reported that the JAK2-STAT3 signalling pathway is primarily responsible for the anti-hyperuricaemic^42^, heart-protective^43^, and renal-protective properties^42^ of curcumin. Curcumin was reported to suppress JAK-STAT signalling and attenuate the inflammatory response in brain glial cells^44^. Furthermore, two small-molecule derivatives of curcumin, FLL32 and FLLL62, were shown to be efficient in treating several different types of cancer including renal cell carcinoma, melanoma, pancreatic cancer, and breast cancer by selectively binding to JAK2 and the STAT3 Src homology-2 domain, which serves a crucial role in STAT3 dimerization and signal transduction^45^. In agreement with these studies, we found that curcumin attenuated neuropathic pain by inhibiting the spinal JAK2-STAT3 cascade. Taken together, these results demonstrated that curcumin inhibits the synthesis of Pro-IL-1β by inhibiting the spinal JAK2-STAT3 cascade.

Recently, 141 patients suffering from neuropathic pain were treated with a formula containing curcumin as one of the major ingredients to test the safety and efficacy of curcumin in the management of neuropathic pain^46^. Therefore, there is an urgent need to better understand the mechanism of action of curcumin before it is used for the treatment of neuropathic pain. The present study showed, for the first time, the differential regulation of spinal IL-1β availability and the maturation that occurs during curcumin-induced anti-nociception following the induction of neuropathic pain in mice. The underlying mechanism might involve the reversal of spinal NALP1 inflammasome aggregation and the inhibition of spinal JAK2-STAT3 cascade activation.

## Methods

### Animals

All of the animal experiments were performed on adult male BALB/c mice that were 6–8 weeks of age and weighed 20–25 g. The mice were supplied by the Experimental Animal Center, Chinese Academy of Sciences, Shanghai. Prior to experimental manipulation, the mice were allowed to acclimate for one week in groups of four mice per cage under controlled conditions (22 ± 1 °C, 6 a.m. to 6 p.m. alternate light-dark cycles) with food and water *ad libitum*. All of the experiments were approved by the Animal Research Welfare Council of School of Basic Medical Science of Fudan University (20140226–086), and conducted strictly in accordance with the National Institute of Health Guide for the Care and Use of Laboratory Animals and the guidelines of the International Association for the Study of Pain^47^. Every effort was made to minimize the number of animals used as well as their suffering.

### Surgical procedures

BALB/c mice were subjected to peripheral neuropathy induced by spared nerve injury as previously described^12^,^48^. In brief, the biceps femoris muscle was exposed under isoflurane anaesthesia (2% isoflurane with oxygen as the carrier gas) delivered via a nose cone. A section was made through the biceps femoris, and the common peroneal and tibial nerves were tightly ligated with 6.0 silk and sectioned distal to the ligation, removing 2–4 mm of the distal nerve stump. For the sham surgery, the sciatic nerve was exposed as described above, but no contact was made with the nerve. Both mechanical threshold and cold sensitivity were tested at the lateral plantar surface of the hind paw.

### Behavioural tests

#### Mechanical stimulation

Mechanical sensitivity was assessed by measuring the withdrawal threshold of the paw ipsilateral to the site of injury in response to mechanical stimuli with Von Frey hairs (Stoelting, Wood Dale, Illinois, USA). The animals were placed in a plastic cage with a wire-net floor and were allowed to habituate 10–15 min before testing began. The animals were also habituated over a period of 2–3 consecutive days, during which time a series of baseline measurements were recorded. A series of eight calibrated Von Frey hairs (0.02, 0.04, 0.07, 0.16, 0.4, 0.6, 1.0, and 1.4 g) were applied to the plantar surface of the hind paw as described previously^12^. Each Von Frey hair was held for approximately 1 to 2 seconds with a 5 minute interval between applications. A trial began with the application of a 0.16 g Von Frey hair. A positive response was defined as a brisk withdrawal of the hind paw upon stimulation. When a positive response to a given hair occurred, the next lower Von Frey hair was applied; and when a negative response occurred, the next higher hair was applied. The test consisted of five more stimuli after the first change in response occurred, and the paw withdrawal threshold was converted to the tactile response threshold using an adaptation of the Dixon up-down paradigm as described previously^49^.

#### Cold stimulation

For the assessment of cold sensitivity, the acetone test was used as described previously^13^,^48^. Approximately 30 min after the Von Frey test, the mice were tested for a paw withdrawal response to a cold stimulus. The test used a 50 μl drop of acetone applied with a syringe fitted with a blunted needle at the centre of the plantar surface of the hind paw ipsilateral to the site of injury, avoiding mechanical stimulation of the paw with the syringe. The total time of the licking/biting of the ipsilateral hind paw was recorded with an arbitrary maximum cut-off time of 60 s. Before testing, the animals were habituated over a period of 2–3 consecutive days, during which time a series of baseline measurements were recorded.

### Intrathecal administration

The intrathecal injections were carried out by lumbar puncture as previously described^50^. Briefly, the mice were anesthetized with isoflurane. Then, drugs (5 μl in volume) were injected into the subarachnoid space of lumbar vertebrae L5 and L6 with a 5 μl syringe (Hamilton, 33-gauge needle). A tail flick indicated that the needle had pierced the dura.

### Drugs

Curcumin was purchased from Sigma (St. Louis, MO, USA). AG490 was purchased from Calbiochem (San Diego, CA, USA). Curcumin and AG490 were dissolved in 20% DMSO and diluted in normal saline. The NALP1 siRNA (sc-63287) and negative control (scrambled siRNA, sc-390133) were purchased from Santa Cruz Biotechnology. To reduce transfection toxicity and efficiently deliver the siRNAs to the spinal cord, the siRNAs were mixed with polyethyleneimine (PEI, Fermentas) according to the manufacturer’s instructions.

### Drug treatment

For repeated curcumin treatment, curcumin or vehicle (20% DMSO) was administered twice daily from day 1 until day 7 after surgery (SNI or sham operation). Fresh curcumin was dissolved in 20% DMSO and diluted to the desired concentration on the day of the experiment.

AG490 was intrathecally injected once daily from day 3 until day 7 after surgery.

For NALP1 or control siRNA treatment, the siRNA was dissolved in RNase-free water at the concentration of 1 μg/μl as stock solution, and mixed with polyethyleneimine (PEI, Fermentas), 10 min before injection, to increase cell membrane penetration and reduce the degradation. PEI was dissolved in 5% glucose, and 1 μg of siRNA was mixed with 0.18 μl of PEI. 5 μl of siRNA (5 μg) was intrathecally injected once a day from day 3 until day 7 after surgery to knockdown NALP1 expression. Gene knockdown in the spinal cord was confirmed by western blotting for NALP1 expression.

### Immunohistochemistry

The mice were deeply anesthetized with 3% isoflurane and perfused intracardially with saline followed by 4% paraformaldehyde in 0.1 M phosphate buffer (PB, pH 7.4). The L4-L5 spinal segments were removed, post-fixed, frozen, sectioned on a freezing microtome (Leica 2000, Germany) at 30 μm thickness, and processed for immunohistochemistry as previously described^48^. The sections were washed three times, blocked with 4% donkey serum in 0.3% Triton X-100 for 1 h at 37 °C, and then incubated in PBS containing 20% normal donkey serum, 0.3% Triton X-100, and a mixture of two primary antibodies overnight at 4 °C. The primary antibodies were mixed as follows: goat anti-IL-1β (1:200; AF-401-NA; R&D, USA) was mixed with mouse anti-glial fibrillary acidic protein (anti-GFAP, astrocyte marker; 1:2000; MA5-12023; Thermo, USA), rabbit anti-pSTAT3 (1:200; #9145; Cell Signaling Technology, USA), or rabbit anti-caspase-1 (1:200; ab17820; Abcam, Cambridge, MA); and rabbit anti-NALP1 (1:200; ab98181; Abcam, Cambridge, MA, USA) was mixed with mouse anti-GFAP (1:2000; Thermo). Following three 15 minute rinses in 0.01 M PBS, the sections were incubated with a mixture of Alexa 594- and Alexa 488-conjugated secondary antibodies (1:1000; Invitrogen, Carlsbad, California, USA) for 1 hour at 37 °C and washed in PBS. All of the sections were cover-slipped using a mixture of 80% glycerin in 0.01 M PBS, and the images were captured using a multiphoton laser point scanning confocal microscopy system (Olympus Fluoview FV1000, Japan; Leica TCS SP5, Germany).

### Western blot analysis

The L4-L5 segments of the spinal cord were homogenized and subjected to SDS-PAGE as previously described^48^. The membranes were incubated with the following primary antibodies at 4 °C overnight: goat anti-IL-1β (1:500; R&D), rabbit anti-caspase-1 (1:500; Abcam), rabbit anti-NALP1 (1:500; Abcam), rabbit anti-ASC (1:500; sc-22514-R; Santa Cruz Biotechnology, Santa Cruz, CA, USA), goat anti-mIL-1β (1:500; sc-23460; Santa Cruz Biotechnology, Santa Cruz, CA, USA), rabbit anti-pSTAT3 (1:500; Cell Signaling Technology), rabbit anti-STAT3 (1:1000; 79D7; Cell Signaling Technology, USA), rabbit anti-pJAK2 (1:500; 3771; Cell Signaling Technology, USA), rabbit anti-JAK2 (1:1000; 3230; Cell Signaling Technology, USA), and mouse anti-β-actin (1:5000; 4967; Cell Signaling Technology, USA). The blots were then washed in TBST and incubated in the appropriate secondary antibody for 1 hour at room temperature. The secondary antibodies were donkey anti-goat lgG-HRP (1:10000; sc-2020; Santa Cruz Biotechnology, CA, USA), donkey anti-mouse lgG-HRP (1:5000; sc-2314; Santa Cruz Biotechnology, CA, USA), or HRP Affinipure Goat Anti-Rabbit lgG (H+L) (1:10000; E030120-01; ErthOx, CA, USA). The western blot images were captured on an ImageQuant LAS4000 mini image analyser (GE Healthcare, Buckinghamshire, UK), and the band levels were quantified using the Image J software, version 1.42q.

### Immunoprecipitation

To determine the composition and association of the proteins in the inflammasome, total protein lysates were extracted from the L4–L5 segments of the ipsilateral spinal cord as previously described^11^. The spinal cord lysates (500 μg) were incubated with 1 μg of an anti-ASC antibody (Santa Cruz Biotechnology, CA, USA) overnight at 4 °C. Then, 50 μl of protein A agarose beads was added to the mixture, and the mixture was incubated at 4 °C for 4 h and centrifuged at 12,000 × g for 60 s. The pelleted beads were washed five times in lysis buffer, resuspended in 2 × loading buffer, and heated at 100 °C for 10 minutes. The immunoprecipitated proteins were analysed by immunoblotting using the following antibodies: rabbit anti-ASC, rabbit anti-NALP1, and rabbit anti-caspase-1. Whole tissue lysate prepared for immunoprecipitation (50 μg) was used as an input, and the non-immunized serum of the same isotype was used as the negative control.

### Real-time PCR

The L4–L6 spinal cord segments of the mice were dissected, frozen in liquid nitrogen, and stored at −70 °C. Total RNA was extracted using Trizol reagent (Invitrogen) according to the manufacturer’s instructions. The amount of RNA was measured using a spectrophotometer. Two micrograms of total RNA was reverse transcribed into cDNA using the M-MLV reverse transcriptase (Promega) and oligo (dT) primers. The PCR reactions were performed using the following oligonucleotide primers for IL-1β (NM_008361.3): upstream primer, 5-actcattgtggctgtggaga-3; and downstream primer, 5-ttgttcatctcggagcctgt-3. The real-time reverse transcription PCR was run on an IQ5 Multicolor Real-Time PCR Detection System (Bio-Rad, Hercules, CA, USA). The expression of IL-1β was normalized to the amount of RNA loaded for each sample using the reference gene β-actin (NM_007393; upstream primer, 5-cctctatgccaacacagtgc-3; and downstream primer, 5-cctgcttgctgatccacatc-3) as an internal standard. The data are presented as the mean ± SEM.

### Statistical analysis

The data are presented as the mean ± standard error (SEM). All of the statistical analyses were performed using the SPSS 19.0 statistical software (SPSS Inc., Chicago, IL). Behavioral data were analyzed by two-way ANOVA (Time × Treatment) followed by Bonferroni *post hoc* test. Western Blot and Real-Time PCR data were analyzed by one-way ANOVA followed by Bonferroni *post hoc* test. Student’s t-test was used when two treatment groups were compared. The density of the specific bands from Western blot gels was measured with a computer-assisted imaging analysis system (Image J, NIH). To quantify the immunoreactive cells in the spinal cord, three or four nonadjacent spinal cord sections were randomly selected from the L4–L5 spinal cord segment. The intensity of IL-1β, GFAP, NALP1, caspase-1 and pSTAT3 staining in the superficial dorsal horn (laminae I–III) were measured by Image J. To determine the degree of double labelling, the number of IL-1β^+^/GFAP^+^, NALP1^+^/GFAP^+^, IL-1β^+^/GFAP^+^ and IL-1β^+^/pSTAT3^+^ double-labelled cells in the dorsal horn (laminae I–III) was also counted by Image J. In all of the statistical analyses, p < 0.05 was considered the criteria for significance.

## Additional Information

**How to cite this article**: Liu, S. *et al*. Curcumin ameliorates neuropathic pain by down-regulating spinal IL-1β via suppressing astroglial NALP1 inflammasome and JAK2-STAT3 signalling. *Sci. Rep.* **6**, 28956; doi: 10.1038/srep28956 (2016).
